# Peptide-Pulsed Dendritic Cell Vaccination Confers Protection Response Against *Paracoccidioides lutzii* in an Experimental Model

**DOI:** 10.3390/jof12100725

**Published:** 2026-09-28

**Authors:** Felipe Antonio Carvalho da Costa, Marcelo Kurgonas de Oliveira, Gabriel Davi Marena, Isabela Souza dos Santos, Maria Clara Pascoal, Eduardo Vieira Farias, Danilo Henrique Ramos, Mel Diniz Sakai, Maira Marrão, Diego Ramos de Moura, Leandro Buffoni Roque da Silva, Joshua Daniel Nosanchuk, Carlos Pelleschi Taborda

**Affiliations:** 1 Laboratório de Fungos Dimórficos Patogênicos, Instituto de Ciências Biomédicas, Universidade de São Paulo, São Paulo 05508-000, Brazilgabmardavi@gmail.com (G.D.M.); mariaclarapascoal@usp.br (M.C.P.); eduviefarias@gmail.com (E.V.F.); meldsakai@usp.br (M.D.S.); maira.marrao@usp.br (M.M.);; 2 Laboratório de Micologia Médica, Instituto de Medicina Tropical de São Paulo, Faculdade de Medicina, Universidade de São Paulo, São Paulo 05403-000, Brazil; 3 Centre for Medical Mycology in Latin America (CMM-LATAM) Unit, São Paulo 05508-000, Brazil; 4 Department of Pediatrics, BC Children’s Hospital, The University of British Columbia, Vancouver, BC V6H 3V4, Canada; lbrsilva87@gmail.com; 5 Covenant HealthCare College of Medicine, Central Michigan University, Mt Pleasant, MI 48859, USA; nosan1j@cmich.edu; 6 Department of Medicine, Division of Infectious Diseases, Albert Einstein College of Medicine, New York, NY 10461, USA

**Keywords:** Paracoccidioidomycosis, dendritic cells, vaccine, peptides

## Abstract

Paracoccidioidomycosis (PCM) is a systemic fungal infection that remains an important public health concern in Brazil. Although the disease is generally responsive to antifungal treatment, the duration of therapy is prolonged and relapses may occur, highlighting the need for alternative strategies and preventative approaches. We have demonstrated that peptide-based vaccines combined with dendritic cell (DC)-based platforms represent a promising strategy to modulate host immune responses against PCM. In this study, we evaluated the immunomodulatory potential of synthetic peptides derived from *Paracoccidioides lutzii* antigens, including the well-characterized P10 peptide from *P. brasiliensis*, integrating in silico prediction, in vitro validation, and in vivo models. In silico prediction utilized available databases and tools to characterize the peptides properties. Lead candidates were synthesized and evaluated for cytotoxicity, demonstrating low toxicity in tested conditions. Stimulation of bone marrow-derived dendritic cells (bmDCs) with the peptides promoted a progressive maturation profile, characterized by increased expression of MHC-II and co-stimulatory molecules, particularly following longer pulsing times. Immunization of mice with peptide-pulsed bmDCs significantly reduced pulmonary fungal burden in infected animals and treatment was associated with lower inflammation and the presence of more compact granulomas, indicating reduced tissue damage. These protective effects correlated with a beneficial cytokine profile, including decreased IL-4, IL-10 and TGF-β levels and increased production of TNF-α, IL-12p70, IL-17A and IL-23, suggesting a shift from a regulatory/Th2 environment toward a protective Th1/Th17 response. Notably, this work is the first to demonstrate that *P. brasiliensis* P10 could confer protection in a *P. lutzii* infection model. Collectively, these findings highlight the potential of peptide-pulsed DC vaccination to reshape pulmonary immunity and improve disease control in PCM.

## 1. Introduction

Paracoccidioidomycosis (PCM) is a systemic endemic mycosis in Latin America, caused by thermodimorphic fungi of the genus *Paracoccidioides* [1,2]. The genus is divided into five species, currently recognized as *P. brasiliensis*, *P. americana*, *P. restrepiensis*, *P. venezuelensis*, and *P. lutzii* [3]. Among these, *P. lutzii* has gained particular attention due to its marked phylogenetic distance, and potential differences in antigenic profile and serological reactivity [4,5,6]. Despite these advances in molecular taxonomy, these species are not easily distinguished with classical routine laboratory methods [7,8].

In 2022, the World Health Organization (WHO) included *Paracoccidioides* in its Fungal Pathogens Priority List [9], underscoring its public health importance. Approximately 80% of reported PCM cases occur in Brazil, where it represents the most prevalent endemic systemic mycosis, with an estimated 3360 to 5600 new cases annually [10,11,12]. PCM ranks among the leading causes of death from endemic infections, with a lethality rate of 3% to 5% [10,11], reaching the highest mortality among mycoses in some states [13]. The disease predominantly affects adult men exposed to fungal propagules through work and environmental activities [2,14], although acute and severe forms may occur in children and pregnant women [15]. Environmental changes, including deforestation, soil disturbance, and climate change, may be reshaping exposure patterns and expanding endemic areas [16,17,18]. Immunocompromised individuals are at increased risk of severe and disseminated disease [19,20,21]. Furthermore, dissemination beyond the primary pulmonary site is common [19,21,22,23]. Although generally effective antifungal agents are available, management of PCM remains challenging because treatment is prolonged (6 months to over 2 years), often complex, and requires long-term follow-up [24,25,26,27]. Effective disease control also depends on a balanced host immune response, with dysregulated T-cell responses contributing to chronicity and relapse [28]. Moreover, even after antifungal treatment, many patients develop chronic pulmonary sequelae, especially fibrosis [29]. Collectively, these factors highlight the urgent need for improved therapeutic strategies, including vaccine development.

The peptide P10 is derived from the immunodominant gp43 antigen of *P. brasiliensis* [30], and has demonstrated protective efficacy in murine PCM models by inducing a robust Th1-associated cytokine responses, which correlate with lower fungal burdens and reduced inflammation [31,32,33], and the protection is improved when P10 immunization is combined with antifungals [34]. To further enhance peptide immunogenicity and bypass the need for conventional adjuvants, dendritic cell (DC)-based immunotherapy was developed as a cellular delivery platform [35], with P10-primed DCs promoting elevated IL-12 levels, reduced IL-4 and IL-10, diminished pulmonary inflammation [36,37], and sustained protection even in previously immunosuppressed mice [38]. However, these previous experiments were carried out using a *P. brasiliensis* strain (Pb18). As briefly mentioned, *P. lutzii* has raised concerns regarding antigenic variability from the other *Paraoccidioides* species, including significant sequence and epitope differences in its gp43 when compared to gp43 of *P. brasiliensis* [39]. The canonical *P. brasiliensis* P10 sequence (QTL**I**AI**HT**LA**I**RYA**N**) differs from its homologous peptide in *P. lutzii* (QTL**A**AI**RA**LA**N**RYA**K**) [40,41]. These observations led us to hypothesize that P10 may have limited cross-species efficacy and applicability in vaccine strategies against PCM caused by *P. lutzii*. In this context, novel immunodominant peptides derived from *P. lutzii* proteins were identified through immunoproteomic approaches and shown to activate DCs, upregulate MHC-II and co-stimulatory molecules, and induce CD4+ and CD8+ T-cell proliferation with a balanced Th1, Th2, and Th17 cytokine profile [42].

Hence, we tested our hypothesis by evaluating the previously identified *P. lutzii*-derived peptides and P10 to support the development of DC-based immunotherapy against PCM caused by *P. lutzii*. We first conducted in silico analyses of all the immunogenic peptides, by reconstructing their three-dimensional structures and its predicted physicochemical properties. Their safety profile was subsequently assessed through in vitro cytotoxicity assays and toxicity evaluation in the *Galleria mellonella* larval model. To enhance immunostimulatory capacity, different pulsing conditions were tested to assessing co-stimulatory molecule expression. Finally, the immunization of DCs pulsed with P10 and the novel peptides was evaluated in a murine model of *P. lutzii* infection.

## 2. Materials and Methods

### 2.1. In Silico 3D Modeling of Peptide Structures

The peptide candidates LBR5, LBR6, LBR8, and P10 were selected for this research based on their performance in the past work of our research group [33,37,38,42]. To ensure clarity and maintain proper attribution, the peptides from *P. lutzii* used in this study were designated as L5, L6, and L8, respectively. Amino acid sequence, protein origin, and reference of each peptide is shown in Table 1.

The amino acid sequences corresponding to the proteins were used for structural modeling. The 3D structures of Hsp70, 40S ribosomal protein S7, and H15 domain-containing protein were obtained from the AlphaFold Protein Structure Database [44]. The gp43 protein structure was modeled based on its sequence deposited in the GenBank Protein database, using ColabFold v.1.5.5 [45]. Structural visualization and highlighting of the modeled peptide sequences were performed using PyMOL v.3.1.6.1 (Schrödinger, New York, NY, USA). The proteins and their respective codes are as follows: Hsp70-like protein—C1GW88 (UniProt); 40S ribosomal protein S7—C1H8U1 (UniProt); H15 domain-containing protein—A0A0A2V091 (UniProt); gp43—AAG36697.1 (GenBank).

### 2.2. Physicochemical Properties of the Peptides

The physicochemical properties were determined using their primary amino acid sequences as input in three online in silico platforms: ExPASy ProtParam (https://web.expasy.org/protparam/, accessed on 20 September 2026), PepCalc (https://pepcalc.com/), and Bachem Peptide Calculator (https://www.bachem.com/knowledge-center/peptide-calculator/, accessed on 20 September 2026). The parameters analyzed included molecular weight, extinction coefficient at 280 nm, isoelectric point (pI), instability index, estimated half-life, net charge at pH 7, predicted solubility, and hydrophilicity. Practical evaluation of solubility was also performed following the manufacturer’s recommendations, in order to verify the computational predictions.

### 2.3. Peptide Synthesis and Preparation

All peptides were synthesized by GenOne Biotechnologies, Rio de Janeiro, Brazil, with a purity of ≥95% and certified endotoxin-free. High-performance liquid chromatography (HPLC) and mass spectrometry (MS) analyses were performed by WatsonBio Sciences, TX, USA, a subcontractor of GenOne Biotechnologies, to ensure proper quality control. Lyophilized peptides L5, L6, and L8 were dissolved in sterile ultrapure water to a final concentration of 2 mg/mL. For the P10 peptide, solubilization was performed by initially adding 20% dimethyl sulfoxide (DMSO—Labsynth, São Paulo, Brazil), followed by dilution in sterile ultrapure water to prepare stock solutions up to 15 mg/mL. Depending on the experimental conditions, peptides were further diluted in appropriate solutions, such as phosphate-buffered saline (PBS—137 mM NaCl, 2.7 mM KCl, 10 mM Na_2_PO_4_, and 1.8 mM KH_2_PO_4_) or cell culture medium. Lyophilized stocks were stored at -20 °C and solution aliquots were kept at 2–8 °C and used within a maximum of one week.

### 2.4. Cytotoxicity in Murine and Human Macrophages

Human THP-1 monocytes and murine RAW 264.7 macrophages were used for cytotoxicity assays. THP-1 cells were maintained in RPMI (Nova Biotecnologia, São Paulo, Brazil) supplemented with 10% FBS (Gibco, Thermo Fisher Scientific, São Paulo, Brazil) and 1% penicillin/streptomycin (Nova Biotecnologia, BR), and differentiated into macrophages with 100 ng/mL phorbol-12-myristate-13-acetate (PMA—Sigma-Aldrich, St. Louis, MO, USA) for 3 days, followed by medium replacement without PMA and a 5-day resting period. RAW 264.7 cells were cultured in DMEM (Nova Biotecnologia, BR) supplemented with 20% FBS and 1% penicillin/streptomycin until appropriate confluence. Adherent cells were harvested using cold medium and gentle scraping, seeded in 96-well plates at 5 × 10^5^ cells/well, and treated with different concentrations of L5, L6, and L8 (5–40 µg/mL). Supplemented medium and 99.9% DMSO were used as viability and death controls, respectively. After 24, 48, and 72 h of incubation at 37 °C and 5% CO_2_, cell viability was assessed using 5 mg/mL of 3-(4,5-dimethylthiazol-2-yl)-2,5-diphenyltetrazolium bromide (MTT—Invitrogen, Thermo Fisher Scientific, Waltham, MA, USA). Formazan crystals were solubilized with DMSO, and absorbance was measured at 570 nm using a microplate spectrophotometer. Viability was expressed as a percentage relative to the untreated control.

### 2.5. Determination of Half-Maximal Inhibitory Concentration (IC50) of P10 in Murine BM Cells and Human Macrophages

THP-1 monocytes were differentiated into macrophages as described previously. Bone marrow (BM) cells were extracted as described below. BM cells were suspended in RPMI 1640 supplemented with 10% FBS, 1% non-essential amino acids, and 10 mM 4-(2-hydroxyethyl)-1-piperazineethanesulfonic acid (HEPES). A suspension of 5 × 10^5^ cells/well was plated in a 96-well plate and incubated at 37 °C with 5% CO_2_. On the following day, the medium was completely replaced with supplemented media, containing the P10 peptide at several concentrations and prepared in the same medium to ensure consistent conditions across wells. Two additional controls were included: a death control, consisting of medium supplemented with 0.5% Triton-X (MSD, Darmstadt, Germany) to establish the minimum viability reference [46]; and a solvent control, corresponding to the highest DMSO concentration present in P10 dilutions (2.73% final concentration). After 24 h, cell viability was assessed using the MTT assay, as previously described. The resulting formazan crystals were solubilized with DMSO, and absorbance was measured at 570 nm using a microplate reader. The IC50 was defined as the P10 concentration that reduced cell viability by 50% relative to the untreated control. The values were determined by nonlinear regression analysis using a four-parameter logistic model, after transformation of concentration data to log10 scale. Absorbance values were blank-subtracted, normalized to the vehicle control (100% viability) and to the death control (0% viability), and then used for plotting the data.

### 2.6. In Vivo Toxicity in a G. mellonella Model

Larvae weighing 150–250 mg were selected from our laboratory colonies maintained at 28 °C and fed pollen and beeswax (Apiários Seiva das Flores, São Paulo, Brazil), weighed, and incubated at 37 °C one day before the assays. For each group, larvae were used in three independent replicates, with *n* value described in the legends. After disinfection with 70% ethanol, larvae were injected via the prolegs using 10 μL Hamilton syringes (Hamilton, Reno, NV, USA) with *P. lutzii* peptides at 2 mg/mL, and P10 at 10 mg/mL. A sham group was used as an untreated control, while larvae injected with PBS or a 99.9% DMSO solution were used as vehicle and toxicity controls, respectively. The number of larvae per group is shown in the graph legends. Larval health was monitored for 5 days using a modified Larval Health Index (LHI), described in the previous study [47], with modifications, assessing activity, melanization, cocoon/pupae formation, and survival. Scores were normalized to a 0–10 scale. For P10, separated data were z-score-normalized and visualized as heatmaps generated using Python (version 3.11) in Google Colaboratory (Google Research).

### 2.7. Murine Experiments

All murine experiments were conducted in accordance with the regulations of the National Council for Animal Experimentation Control (CONCEA) and the Ethics Committee for the Use of Animals (CEUA) of the Instituto de Ciências Biomédicas da Universidade de São Paulo (ICB-USP), registered in project nº. 2373190722, approved on 26 October 2022. BALB/c mice, specific pathogen-free, male, aged 7 to 9 weeks, were bred in the Central Animal Facility at FMUSP. The experiments were performed in the Animal Facility of ICB-II and the Laboratório de Fungos Dimórficos Patogênicos at ICB-II. The animals were housed in cages under a 12 h light/dark cycle, with ad libitum access to water and food, and handled according to good laboratory animal management practices. Euthanasia was performed by an intraperitoneal (i.p.) injection containing a solution of 300 mg/kg of ketamine and 30 mg/kg of xylazine (both from Ceva Brasil, São Paulo, Brazil).

### 2.8. Generation of Bone Marrow-Derived Dendritic Cells (bmDCs)

Standardization experiments were conducted to ensure the reproducibility of bmDC-based assays, following the methods of previous studies [42,48,49]. Mice were euthanized and femurs, tibias, and humeri were collected under aseptic conditions. After removing surrounding tissues, bones were washed, and BM cells were flushed with RPMI medium supplemented with 10% FBS and 1% penicillin/streptomycin. The cell suspensions were filtered through a 70 µm cell strainer, centrifuged, and subjected to red blood cell lysis (12 mM NaHCO_3_, 155 mM NH_4_Cl, and 0.1 mM EDTA). The resulting BM cells were then suspended in complete medium and counted for subsequent assays. For the differentiation into bmDCs, 1.5 × 10^6^ cells/well were plated in a 12-well plate cultured in RPMI medium supplemented with 10% FBS, 1% non-essential amino acids, 1% penicillin/streptomycin, 30 ng/mL GM-CSF, and 15 ng/mL IL-4 at 37 °C and 5% CO_2_. Medium replenishment was performed on days 2 and 4. By day 6, a population containing bmDCs was obtained and used in subsequent experiments.

### 2.9. Activation Assay of bmDCs

To evaluate bmDC activation, cells were stimulated on day 6 of differentiation with peptides or control stimuli. Unstimulated bmDCs maintained under standard culture conditions served as the non-pulsed control, whereas bmDCs stimulated with 1 µg/mL lipopolysaccharide (LPS—Thermo Fisher Scientific, USA) served as the positive activation control. Peptide-pulsed groups consisted of bmDCs incubated with 10 µg/mL of the L5, L6, or L8 peptide individually. An additional group was pulsed with a combination of these peptides (3.33 µg/mL each; LX) to evaluate potential synergistic effects. Another group was stimulated with P10 at 433 µg/mL (255 µmol/L). These concentrations were used as defined by previous results [37,38,42]. Depending on the experimental design, cells were incubated with the respective stimuli for either 6 or 22 h. Culture supernatants were then collected and stored at −20 °C until cytokine quantification.

### 2.10. Immunophenotyping of bmDCs

Flow cytometry staining was performed as described [42], with minor modifications. Briefly, bmDCs were gently collected using ice-cold FACS buffer, centrifuged, and resuspended in FACS buffer solution (PBS, 1% FBS, 2 mmol L-1 EDTA, and 0.1% sodium azide). Cells were counted and around 10^6^ cells/well were transferred to V-bottom 96-well plates for staining. Surface staining was performed using the fluorochrome-conjugated antibodies: MHC-II-PerCP (M5/114), CD11c-BV711 (HL3), Ly6c-BV605 (HK1.4.rMAb), CD40-APC (3/23), CD80-PE (16-10A1), CD86-FITC (GL1) (all from BD Biosciences, Franklin Lakes, NJ, USA). Cell viability was assessed using Violet Live/dead (Invitrogen, Thermo Fisher Scientific, USA). Cells were incubated with an antibody cocktail for 30 min, protected from light, followed by washing with FACS buffer. To establish the dead-cell control for viability staining, an aliquot of cells was incubated at 60 °C for 1 h before staining. Following surface staining, cells were fixed with 4% paraformaldehyde for 15 min, washed and resuspended in FACS buffer. Samples were stored at 4 °C, protected from light, until acquisition. Compensation was performed using beads stained with the corresponding fluorochrome-conjugated antibodies. Samples were acquired on a BD LSRFortessa™ Cell Analyzer, and data were processed using FlowJo™ v.10 software (BD Biosciences). Expression of activation markers was quantified as median fluorescence intensity (MFI).

### 2.11. In Vitro Pulsing of bmDCs and Immunization

Following BM cell extraction and differentiation into bmDCs, cells were plated in 6-well plates on day 6 of differentiation and pulsed with peptides L5, L6, or L8 (10 µg/mL). An additional group was pulsed with an equal mixture of the three peptides, designated LX (3.33 µg/mL of each peptide). Control groups consisted of unpulsed cells and bmDCs pulsed with P10 (433 µg/mL). After 24 h of pulsing, the cells were harvested as described above, washed, suspended in PBS, counted, and assessed for viability using trypan blue exclusion. The cells were then adjusted to a final concentration of 3 × 10^6^ viable cells/mL for each condition [37,38]. All conditions had been previously standardized and showed cell viability above 80%. Cells suspensions were then loaded into syringes with 31G needles and kept at 4 °C until administration into the animals on the same day. Mice were immunized with a single 100 µL subcutaneous injection 7 days prior to *P. lutzii* intranasal (i.n.) infection, as in the previous study [36].

### 2.12. Fungal Strain

The *P. lutzii* strain (Pb01), used for the in vivo infection experiments, was kindly provided by the Laboratório de Micologia Médica (LIM-53) do Instituto de Medicina Tropical de São Paulo (IMT), a national reference laboratory that maintains the mycological collection “Prof. Carlos da Silva Lacaz” and performs regular genetic quality control and preservation of fungal isolates. Yeast-phase cultures were maintained on Fava Neto agar at 37 °C. Prior to infection, yeast cells were transferred to Erlenmeyer flasks containing Brain Heart Infusion (BHI) broth supplemented with 4% FBS, 4% glucose (Inlab, São Paulo, Brazil), and 1% penicillin/streptomycin. Cultures were incubated at 36 °C under agitation (150 rpm) for 5–7 days before infection. Fungal cells were then centrifuged at 4000 rpm for 5 min, washed with PBS, and passed through a 70 µm cell strainer. Yeast suspensions were subsequently transferred to syringes and sheared by alternating passages through 21G and 26G needles to disrupt aggregates. Cell viability was assessed using trypan blue solution, and yeast counts were performed in a Neubauer chamber. Only inocula with viability > 80% were used in the experiments.

### 2.13. Murine Infection

Mice were anesthetized by i.p. injection containing 70 mg/kg ketamine and 15 mg/kg xylazine [50]. After confirmation of anesthesia, 25 µL of a yeast suspension containing 5 × 10^6^ yeasts/50 µL in PBS were delivered into each nostril. Thirty days post-infection, mice were weighted, euthanized, and lungs and spleen were collected for downstream analysis. Individual organ weights were normalized to the corresponding total body weight of each mouse, to minimize corporal body variation.

### 2.14. Quantification of Fungal Burden

Lungs and spleens were weighed and then subjected to homogenization using the gentleMACS™ Dissociator (Miltenyi Biotec, Bergisch Gladbach, Germany). Fungal burdens were quantified by plating 200 µL of each homogenate into BHI agar supplemented with 4% FBS, 4% glucose, 5% Pb192 culture filtrate, and 1% penicillin/streptomycin. Plates were incubated at 35 °C. Colony-forming units (CFUs) were recorded for up to 14 days.

### 2.15. Quantification of Cytokines

Lung homogenates were centrifuged at 2000 rpm for 5 min to remove most tissue debris, then supernatants were collected and stored at −20 °C until analysis. Cytokine concentrations of IFN-γ, TNF-α, TGF-β, IL-1β, IL-4, IL-6, IL-10, IL-12p70, IL-17A and IL-23 were quantified using commercially available ELISA kits (BD Biosciences, USA; Thermo Fisher Scientific, USA), according to the manufacturer’s instructions. Briefly, samples and standards were added to antibody-coated plates, incubated with detection antibodies and substrate solution, and the reaction was stopped according to the protocol. Absorbances were measured at 450 nm using a microplate reader, and cytokine concentration was calculated from the corresponding standard curves. Data from supernatants were subsequently analyzed using Python, in which results were transformed by z-score normalization and visualized as a heatmap to compare cytokine expression patterns among experimental groups.

### 2.16. Histological Analysis

Lung sections were collected for histological analysis. Tissue processing and slide preparation were performed by the Histological Techniques Core Facility of ICB-II. Lung fragments were fixed in 10% buffered formalin and then transferred to 70% ethanol. After paraffin embedding, thin sections were obtained, mounted on glass slides, and stained with hematoxylin and eosin (H&E). Histological evaluation was performed using conventional light microscopy.

### 2.17. Statistical Analysis

The software GraphPad Prism 10 was used for statistical analysis, with data procedures based on published research [51]. The evaluation and statistical methods used are described in the legend of each graph, including data expression, comparisons between experimental groups and method tested. Differences were considered statistically significant when *p* < 0.05.

## 3. Results

### 3.1. Peptides Display Distinct Physicochemical Profiles

The physicochemical properties of the selected peptides were predicted in silico on their primary amino acid sequences. Although some minor discrepancies were observed among the prediction tools, only one database was selected for each parameter to ensure consistency in the analysis. The predicted properties are summarized in Table 2.

Solubility results are shown in Table 3. Between databases, findings obtained from PepCalc and Bachem were consistent, indicating that the predicted solubility profiles were aligned with negative average hydrophobicity values of the peptides. Still, regarding in silico solubility predictions, PepCalc also indicated “poor water solubility” for peptides L5, L8, and P10. However, by following the manufacturer’s recommendations, practical experiments revealed that L5 and L8 reconstituted in ultrapure water had complete solubilization (absence of precipitates), in contrast to the PepCalc prediction. P10 was successfully solubilized using DMSO followed by ultrapure water, with no visible precipitation under optimized conditions. However, at certain concentrations, precipitate formation was clearly observed for this peptide.

### 3.2. AlphaFold-Based Modeling Reveals the Structure of the Selected Peptides

To further characterize structural features of the selected peptides, we first generated three-dimensional models of the four source proteins and their peptides using AlphaFold and ColabFold, followed by structural visualization in PyMOL. Predicted structures indicated that the full-length proteins predominantly exhibited α-helical secondary structure. Notably, the regions corresponding to the selected peptides were located within predicted α-helical segments of the proteins, as highlighted in Figure 1.

### 3.3. Peptides Do Not Exhibit Cytotoxic Effects on Murine or Human Immortalized Macrophages

To establish the in vitro safety profile of the selected *P. lutzii* peptides, we next evaluated their effects on cell viability. Cytotoxicity was evaluated by the MTT assay in RAW 264.7 and THP-1 macrophages exposed to *P. lutzii* peptides at different concentrations and time points. As shown in Figure 2, no significant cytotoxic effects were observed in THP-1 cells compared with the media control. Similarly, RAW 264.7 macrophages maintained high viability across all conditions. Although a statistically significant reduction was detected for peptide L8 at 20 µg/mL, mean viability remained above 75%, and higher concentrations did not result in significant differences compared with the control group.

### 3.4. Determination of IC50 Values for Peptide P10 Reveals Cell-Type-Specific Differences

Given the physicochemical characteristics of P10, including its limited solubility, requirement for potentially toxic solvents, and the tendency to form precipitates, we next determined the in vitro IC50. Multiple dilutions and solubilizations were evaluated, which led to a maximum concentration of 2048 µg/mL. This concentration was subsequently used as the starting point for serial dilutions. Importantly, the corresponding DMSO concentration was limited to 2.73%, allowing us to evaluate the intrinsic cytotoxicity of P10 while minimizing solvent-related undesirable effects. Using THP-1 macrophages and murine BM cells, it was possible to characterize the IC50 values of peptide P10 in both cellular populations. The results demonstrated a clear difference in cytotoxicity between the two cell populations, as shown in Figure 3 and Table 4.

The IC50 of P10 was 2.35-fold lower in BM cells than in THP-1 macrophages, indicating that BM cells are more sensitive to P10-induced cytotoxicity and highlighting a cell type-dependent response.

### 3.5. No Toxic Effects of Peptides Were Observed in a G. mellonella Model

A *G. mellonella* model was used to evaluate in vivo toxicity of the peptides. Larval survival curves and LHI scores were determined to characterize peptide toxicity, and the results are presented in Figure 4.

As expected, peptides did not induce detectable toxicity, with no significant differences in survival observed among all groups when compared with the sham and PBS control groups, where both showed survival rates above 95%. In contrast, the DMSO control group exhibited nearly 100% larval mortality within the first 24 h, confirming the expected toxicity of the solvent (Figure 4A). The LHI assessment is presented in Figure 4B, which considered parameters such as activity, cocoon formation, web production, melanization, and survival. Scores ranging between 8 and 10 indicate a healthy larvae group, whereas values below 6 reflect progressive deterioration of larval health. The results showed that larvae exposed to *P. lutzii* peptides consistently presented health indices above 8, indicating that the tested peptides did not impact larval health.

P10 was evaluated separately at a higher concentration (10 mg/mL) using the same *G. mellonella* model. As shown in Figure 4C, P10 did not significantly reduce larval survival, although an approximately 20% decrease was observed compared with the control groups. Similarly, LHI scores remained comparable among the experimental groups (Figure 4D), despite a tendency specifically toward increased melanization in P10-treated larvae. To further investigate this observation, daily melanization scores extracted from the LHI assessment were visualized as a heatmap (Figure 4E). Although larvae injected with P10 exhibited higher melanization scores than the control groups, these differences were not statistically significant.

### 3.6. Prolonged Peptide Exposure Enhances Murine bmDC Activation and Receptor Expression

To evaluate the immunomodulatory effects of the peptides, bmDCs were stimulated for 6 h with each peptide individually or with a mix of all *P. lutzii* peptides (LX group). Non-stimulated cells and LPS-treated bmDCs were included as controls. As shown in Figure 5, exposure to the *P. lutzii*-derived peptides significantly increased MHC-II expression compared with non-stimulated cells. A similar pattern was observed for CD80 and CD86, indicating enhanced bmDC activation. In contrast, CD40 expression was not significantly altered by any peptide or even LPS stimulation, likely reflecting the relatively short stimulation period. Although P10 increased MHC-II expression, this effect did not reach statistical significance. However, P10 significantly upregulated CD80 and CD86 expression.

To investigate whether prolonged peptide exposure enhanced bmDC activation, cells were incubated with the peptides for 22 h, based on the in silico peptide half-life predictions (Table 2). The results are shown in Figure 6. Compared with the 6 h stimulation, a general increase in the expression of all activation markers was observed after 22 h of peptide exposure. MHC-II expression showed significant upregulation compared to the non-pulsed group. CD40 expression was significantly increased in all peptide-stimulated groups, with the most pronounced effects observed in cells stimulated with peptides P10, L8 and LX. CD80 and CD86 were also significantly upregulated in response to all peptides. Notably, bmDCs stimulated with the peptide combination (LX group) exhibited a slightly greater increase when compared with the conditions with peptides alone, suggesting that combining different epitopes may enhance bmDC activation.

We next characterized the cytokine profile of bmDCs following stimulation by quantifying cytokine levels in the culture supernatants, as shown in Figure 7. As expected, stimulation with LPS induced a strong pro-inflammatory response, characterized by high IL-6 production. In contrast, peptide P10 promoted a marked increase in IL-10 levels, while also inducing moderate production of IL-12p70. The remaining peptides elicited more modest cytokine responses overall. Among them, the L8 group was associated with relatively higher IL-12p70 production compared to the other peptides, whereas L5 and L6 groups showed low to moderate levels of the cytokines evaluated. Unexpectedly, the LX group displayed minimal cytokine induction, with levels close to those observed in the unstimulated control. Notably, IL-1β and TNF-α levels were below the limit of detection under all experimental conditions.

### 3.7. Pulsed bmDCs Reduce Pulmonary Fungal Burden in P. lutzii-Infected Mice

To evaluate the in vivo immune response induced by the peptide-pulsed bmDC vaccine, a prophylactic mouse model of *P. lutzii* infection was established, in which mice were immunized with peptide-pulsed bmDCs prior to fungal challenge, as illustrated in Figure 8A.

Fungal burden analysis demonstrated that prophylactic immunization with peptide-pulsed bmDCs significantly reduced the number of viable *P. lutzii* yeasts recovered from the lungs when compared with the infected control group (Figure 8B). Unpulsed bmDCs also significantly reduced pulmonary fungal burden relative to infected controls, indicating that bmDCs alone produce a beneficial effect by also reducing the fungal burden. However, peptide-pulsed bmDCs provided greater protection than unpulsed bmDCs. Among the vaccinated groups, DC-L8 and DC-LX were particularly effective, with several mice showing no detectable fungal growth in the lungs. Surprisingly, P10-pulsed bmDCs also significantly reduced the pulmonary fungal burden following *P. lutzii* infection. No fungal colonies were recovered from the spleens of any experimental group.

Consistent with the reduction in fungal burden, prophylactic immunization also reduced lung weight compared with the infected control group (Figure 8C), suggesting attenuation of pulmonary inflammation. As expected, infected mice exhibited significantly heavier lungs than non-infected animals, reflecting the inflammatory response associated with infection. In contrast, spleen weight did not differ significantly among the experimental groups (Figure 8D).

### 3.8. Peptide-Pulsed bmDCs Increase Th1/Th17- and Decrease Th2/Treg-Associated Cytokines in P. lutzii-Infected Lungs

The pulmonary cytokine profile was also evaluated to further characterize the immune response induced by prophylactic immunization, and the results are presented in Figure 9.

TNF-α levels remained at basal levels in infected mice that did not receive peptide-pulsed bmDC immunization. However, all groups immunized with peptide-pulsed bmDCs prior to infection exhibited significant increase in TNF-α levels, with the greatest increases observed in the DC-P10 and DC-LX groups when compared with the infected-only control group (Figure 9B). In contrast, no significant differences were observed in IFN-γ or IL-1β among the experimental groups (Figure 9C,D). On the other hand, infection induced a marked increase in IL-4, IL-6, IL-10, and TGF-β compared with non-infected animals, indicating the establishment of an inflammatory environment associated with regulatory and Th2-type responses (Figure 9E–G,K). Prophylactic immunization significantly reduced the levels of these cytokines, suggesting that peptide-pulsed bmDC vaccination modulated the pulmonary inflammatory response. Conversely, IL-12p70 production was significantly enhanced in immunized mice compared with infected controls (Figure 9H). Likewise, IL-17A and IL-23 levels were significantly increased following immunization, whereas infected animals exhibited cytokine levels comparable to those of non-infected controls (Figure 9I,J). These findings suggest that bmDC vaccination promotes a protective immune response characterized by increased IL-12p70 and Th17-associated cytokine production while limiting excessive Th2 and regulatory-associated cytokine responses.

### 3.9. Vaccination with Peptide-Pulsed bmDCs Promotes Organized Granulomatous in the Lungs

Histopathological evaluation revealed preserved pulmonary architecture in non-infected animals, with normal alveolar spaces and absence of infiltrates (Figure 10A). In contrast, infected animals presented intense inflammatory infiltrates and poorly organized granulomatous lesions within the pulmonary parenchyma (Figure 10B). In vaccinated groups, granulomatous structures with a more compact and organized morphology were observed (Figure 10C–H). In addition, inflammatory infiltrates composed of polymorphonuclear and mononuclear cells were detected surrounding the granulomas. In general, peptide-based vaccination was relatively associated with the formation of more structured granulomatous lesions compared with the non-vaccinated infected group.

## 4. Discussion

DC-based platforms have emerged as a promising strategy for the development of therapeutic and prophylactic vaccines, particularly in infectious diseases and cancer [52,53,54]. As professional antigen-presenting cells, DCs efficiently process and present antigens to T lymphocytes, bridging innate and adaptive immunity and promoting antigen-specific immune responses [55]. However, the success of this approach relies not only on the cellular platform itself but also on the careful selection and characterization of the antigens used to pulse these cells. In this context, peptide antigens represent attractive candidates because of their low production cost and high purity [56]. Nevertheless, candidate peptides should undergo comprehensive characterization before their application in cellular vaccines, including in silico prediction of their immunogenic potential [57,58] and evaluation of their physicochemical properties, since inadequate characteristics may compromise peptide handling, stability, antigen presentation, and cellular viability during in vitro pulsing [59].

In this work, the four peptide candidates were first characterized in silico by evaluating their physicochemical properties, which revealed distinct patterns and formulation requirements. To gain deeper insights into their structural characteristics, high-resolution three-dimensional models of their corresponding source proteins were generated using AlphaFold and ColabFold, followed by visualization in PyMOL (Figure 1). AlphaFold, a state-of-the-art deep learning approach, predicts protein structures with accuracy comparable to experimental methods and surpasses previous computational algorithms [44,45]. Since *P. lutzii* peptides were selected based on their binding to MHC-II [42,60], structural modeling revealed that all peptides contain small α-helical segments, suggesting that this feature may influence positively immune interactions, also correlated with high immunogenicity as shown in previous studies [61,62].

As macrophages represent one of the critical cell populations to interact with administered vaccine elements, we used this cell type to evaluate the safety of the peptides. Both THP-1 and RAW 264.7 macrophages demonstrated high viability when exposed to the *P. lutzii* peptides, which suggests the absence of major cytotoxic effects and reinforces their potential suitability for continued preclinical development (Figure 2).

Since its identification, the peptide P10 has been widely explored as a vaccine candidate for PCM [31,32,33,37,38,42,63,64,65,66]. However, technical limitations have been reported, particularly regarding its low solubility (Table 3). Previous studies also demonstrated that P10 has a propensity to form amyloid fibrils and steric zipper structures at physiological pH [41], which may explain the precipitation observed during handling and represents an undesirable feature for vaccine formulations. However, these studies did not evaluate the cytotoxicity of P10 using biological models. In other study, the cytotoxicity of formulations containing 2 mg/mL of P10 encapsulated with nanoparticles was evaluated, and no cytotoxic effects were observed in J774.16 macrophages, with cell viability remaining above 50% after 72 h of incubation [65]. However, no firm conclusions could be made regarding the cytotoxic dosage of P10 when used alone. For that reason, we decided to determine the IC50 of P10 in THP-1 macrophages and murine BM cells. Our results revealed inhibitory effects only at high concentrations (Figure 3 and Table 4) and a greater sensitivity of primary BM cells. This difference likely reflects the lower resistance of primary cells to toxic stimuli [67], as well as intrinsic properties of THP-1 cells, which may exhibit increased resistance to death due to leukemia-associated adaptations [68]. Together, these observations provide important parameters for defining appropriate P10 concentrations in DC-based pulsing strategies.

The use of *G. mellonella* larvae represents a practical intermediate model to assess compound safety before mammalian studies [69,70]. In this work, neither survival curves nor larval health scores indicated relevant toxicity associated with the *P. lutzii* peptides (Figure 4A,B). Similarly, P10 did not significantly affect survival or LHI (Figure 4C,D), despite this concentration being considerably higher than those previously used in murine experiments [31,32,33,37,38,42,63,64,65]. The modest increase in melanization observed in the P10 group (Figure 4E) most likely reflects immune activation rather than toxicity, consistent with the immunogenic nature of gp43-derived peptides reported in other studies [30,43].

The immunomodulatory capacity of the peptides was further explored through their effects in bmDC activation assays. Extended peptide exposure promoted a progressive maturation profile, characterized by increased expression of MHC-II and co-stimulatory molecules over time (Figure 5 and Figure 6) complemented by distinct cytokine production patterns (Figure 7). These activation effects in bmDCs have been previously described for P10 [37]; however, the impact of extended pulsing duration had not been investigated. Additionally, the CD40 expression exhibited distinct kinetics compared with CD80 and CD86. These molecules, members of the B7 family, are typically upregulated early during DC activation [55,71]. On the other hand, CD40 is a member of the TNF receptor superfamily, which contributes to the later stages of antigen-presenting cell (APCs) maturation. Engagement of CD40 by its ligand expressed on T-cells induces signaling pathways that promote APC maturation, enhance CD80/CD86 expression, stimulate IL-12 production, and support CD8+ T-cell expansion [72,73]. Therefore, CD40 expression represents an important marker when evaluating the activation state of DCs. Our group is currently extending these findings to human donor-derived DCs to investigate the immunogenic potential of these peptides in the context of PCM and to explore chitosan nanoparticle-based formulations as a strategy to enhance their immunogenicity [74].

Effective immunity against PCM depends on the formation of organized pulmonary granulomas composed predominantly of phagocytic cells [75]. In susceptible hosts, dysregulated immune responses characterized by predominant Th2/Treg cytokines impair Th1/Th17 immunity, favoring fungal dissemination [28,34,38,76,77]. In this context, DC-based vaccines represent a promising strategy to promote protective immune responses, improve disease control, and potentially reduce the risk of relapse by reinforcing antigen-specific cellular immunity [78,79]. In this study, prophylactic immunization with peptide-pulsed bmDCs significantly reduced pulmonary fungal burden of infected animals (Figure 8B). This protection was accompanied by lower lung-to-body weight ratios (Figure 8C), more compact granulomas formation, and increased infiltration of polymorphonuclear and mononuclear cells (Figure 10), findings consistent with improved control of pulmonary infection and reduced tissue damage. Taken together, these results suggest that peptide-pulsed bmDC vaccination is capable not only of restricting fungal proliferation but potentially limit the *P. lutzii* consequences of infection in the lung environment.

Infected non-immunized mice displayed elevated levels of IL-4, IL-10 and TGF-β (Figure 9), consistent with a regulatory/Th2 immune environment that is frequently associated with impaired fungal clearance and dissemination of *Paracoccidioides* [28]. In contrast, bmDC-immunized mice groups showed reduced production of these cytokines in the lungs, indicating that prophylactic immunization regulates the excessive regulatory response typically induced during infection and promotes a more balanced immune response. Concurrently, cytokines associated with antifungal protection were enhanced in immunized mice groups. Increased TNF-α and IL-12p70 levels suggest activation of innate immune mechanisms and support for Th1-type responses, both of which are important for macrophage activation and granuloma organization during PCM immune response [80,81]. In parallel, the increased production of IL-17A and IL-23 suggests activation of Th17 cells, which contributes to fungal control through the recruitment activation of neutrophils [82,83,84]. The absence of IL-17A upregulation in infected non-immunized animals further suggests that peptide-pulsed bmDCs were essential for driving this response. Collectively, these findings indicate that this approach reshaped the pulmonary immune environment, shifting it from a regulatory/Th2-dominant profile toward a protective Th1/Th17-associated response.

Importantly, these findings are consistent with previous work from our group evaluating the same *P. lutzii*-derived peptides. In that study, peptide immunization induced antigen-specific lymphocyte proliferation, particularly of CD4+ T-cells in response to L5 and L6, while L8 stimulated both CD4+ and CD8+ T-cell responses [42]. Moreover, bmDCs pulsed with these peptides efficiently promoted CD4+ T-cell proliferation, confirming their ability to present these antigens and activate adaptive immunity. Cytokine analyses of lymphocyte cultures further demonstrated increased production of IFN-γ, TNF-α and IL-17A, especially after stimulation with L8, supporting the induction of a Th1/Th17 immune profile [42]. In our earlier work, a similar cytokine profile was also observed following co-culture of lymphocytes with peptide-pulsed bmDCs, indicating that DC-mediated antigen presentation effectively primed T-cells. Our current results significantly extend these prior observations by demonstrating that our immunization scheme not only promotes protective cytokine patterns in the lungs after *P. lutzii* infection, but it also contributes to a reduced fungal burden, preserved pulmonary architecture, and attenuation of inflammatory damage. Collectively, these findings strengthen the evidence that these peptides act by eliciting antigen-specific cellular immunity capable of controlling PCM in mice. While direct comparisons between studies should be made cautiously due to methodological differences, such as modifications in the timing of peptide-pulsing protocol and experimental design, the overall findings consistently support protective potential of these peptide-pulsed bmDC immunization.

Modulation of MHC-II-mediated peptide presentation is an important determinant of vaccine-induced protective responses [55,85]. Subunit vaccines that contains highly defined epitopes are attractive for vaccine development, particularly for vulnerable populations, as they may reduce exposure to unnecessary microbial components and, consequently, the risk of adverse effects [86,87]. However, vaccines with limited number of epitopes may also provide less diversity of antigenic targets and potentially fail to elicit a comprehensive immune response [87]. Based on this, we explored a multi-epitope approach using a combination of the three previously identified *P. lutzii* peptides. Regarding this, our results showed no significant differences in pulmonary fungal burden compared with the individual peptide-pulsed bmDC groups (Figure 8B; Supplementary Table S1). Although combining multiple epitopes could theoretically broaden antigen-specific immune responses and provide more comprehensive recognition of the pathogen, our current findings do not allow us to determine whether this multi-epitope formulation provides an advantage or disadvantage over individual peptides. Further studies evaluating the specificity beyond immunomodulatory effects, and functionality of the immune response elicited by individual versus combined peptides will be necessary to clarify the potential benefits of this strategy.

Several limitations of the study should be acknowledged. The chronic PCM model was evaluated exclusively in male BALB/c mice to maintain consistency with previous studies, including those evaluating P10, and because female sex hormones may influence disease outcome and the biology of thermodimorphic fungi [88], potentially being one of many reasons of higher prevalence of PCM in men [10,12]. Future studies should therefore investigate sex-related differences in vaccine-induced protection. In addition, only one *P. lutzii* strain (Pb01) was evaluated, and other *Paracoccidioides* species should be investigated. The intranasal infection route was selected based on established experimental PCM models [82,89], although other routes, such as intratracheal infection, may be technically challenging due to fungal aggregation. Finally, this study evaluated only prophylactic vaccination, and the therapeutic efficacy of peptide-pulsed DCs in animals with established infection would be highly relevant for future clinical studies.

Although P10 is derived from *P. brasiliensis* gp43 and has previously been shown to confer protection against *P. brasiliensis* [31,32,33,37,38,42,63,64,65], its protective potential against *P. lutzii* had not been established before. This is particularly relevant given the differences between the gp43 proteins of the two species. Here, we demonstrate that P10-pulsed bmDCs confer significant protection against experimental *P. lutzii* infection. Furthermore, we demonstrate that the previously identified *P. lutzii*-derived peptides also confer protective immune responses in infected mice. Ongoing studies are aimed at further understanding the specificity and mechanism underlying these peptide-induced immune responses. Overall, our findings reinforce the potential of peptide-pulsed bmDC vaccination strategies to modulate the pulmonary immune response toward a protective Th1/Th17-associated cytokine profile, resulting in reduced fungal burden, preservation of lung architecture, and attenuation of pulmonary inflammation during experimental *P. lutzii* infection.

## 5. Conclusions

This study demonstrates that peptide-pulsed bmDC vaccination represents a promising immunomodulatory strategy against *P. lutzii* infection. The evaluated peptides displayed suitable physicochemical characteristics and favorable safety profiles, showing low cytotoxicity in different cells types in vitro models and an absence of significant toxicity in a *G. mellonella* model. In addition, peptide stimulation promoted bmDCs maturation, characterized by increased expression of antigen presentation and co-stimulatory molecules. In vivo, bmDC immunization significantly reduced pulmonary fungal burden, limited lung inflammation and tissue damage, and reshaped the molecular environment toward a protective Th1/Th17-oriented cytokine response while reducing regulatory cytokines associated with disease progression. Importantly, this work provides the first evidence that the classical P10 peptide, originally described for *P. brasiliensis*, can also confer protection against *P. lutzii* infection in mice. Together, these findings highlight the potential of peptide-based DC vaccines to modulate antifungal immunity and support further investigation of peptide candidates as vaccine tools for PCM.

## Figures and Tables

**Figure 1:**
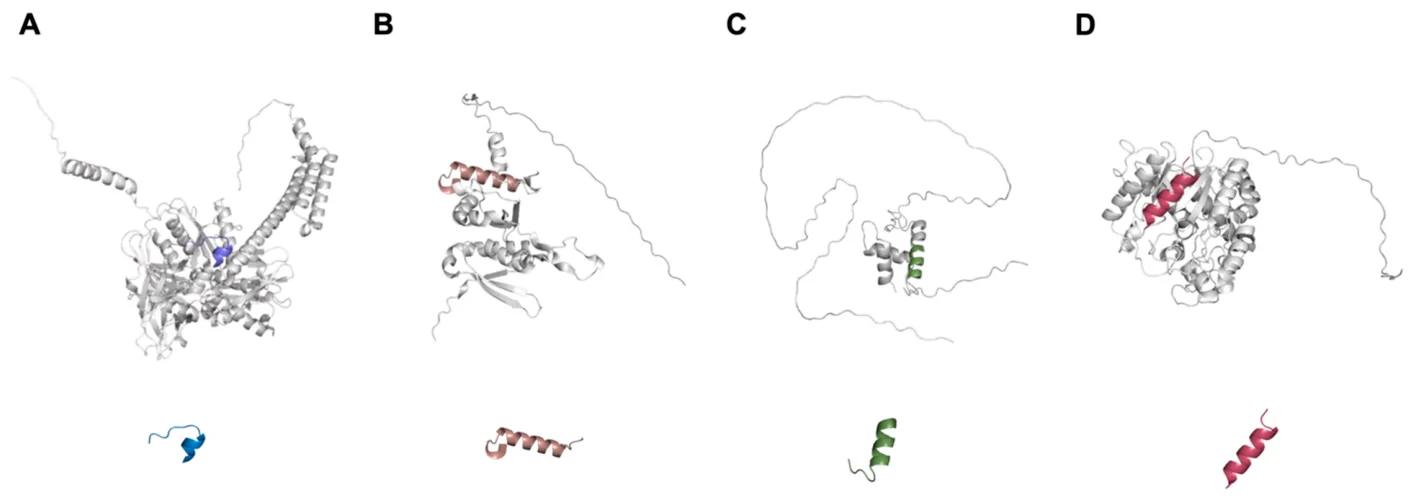
In silico 3D structure of the proteins of origin and their corresponding peptides. The base structure of the protein from *P. lutzii* (Pb01) was collected from the AlphaFold Protein Structure Database. For gp43 of *P. brasiliensis* (Pb339), the structure was created with ColabFold. All final structures were modeled and had the peptide sequence highlighted in PyMOL. (**A**) Hsp70-like protein (gray) and L5 sequence (blue); (**B**) 40S ribosomal protein S7 (gray) and L6 sequence (pink); (**C**) H15 domain-containing protein (gray) and L8 structure (green); (**D**) Gp43 (gray) and P10 structure (purple).

**Figure 2:**
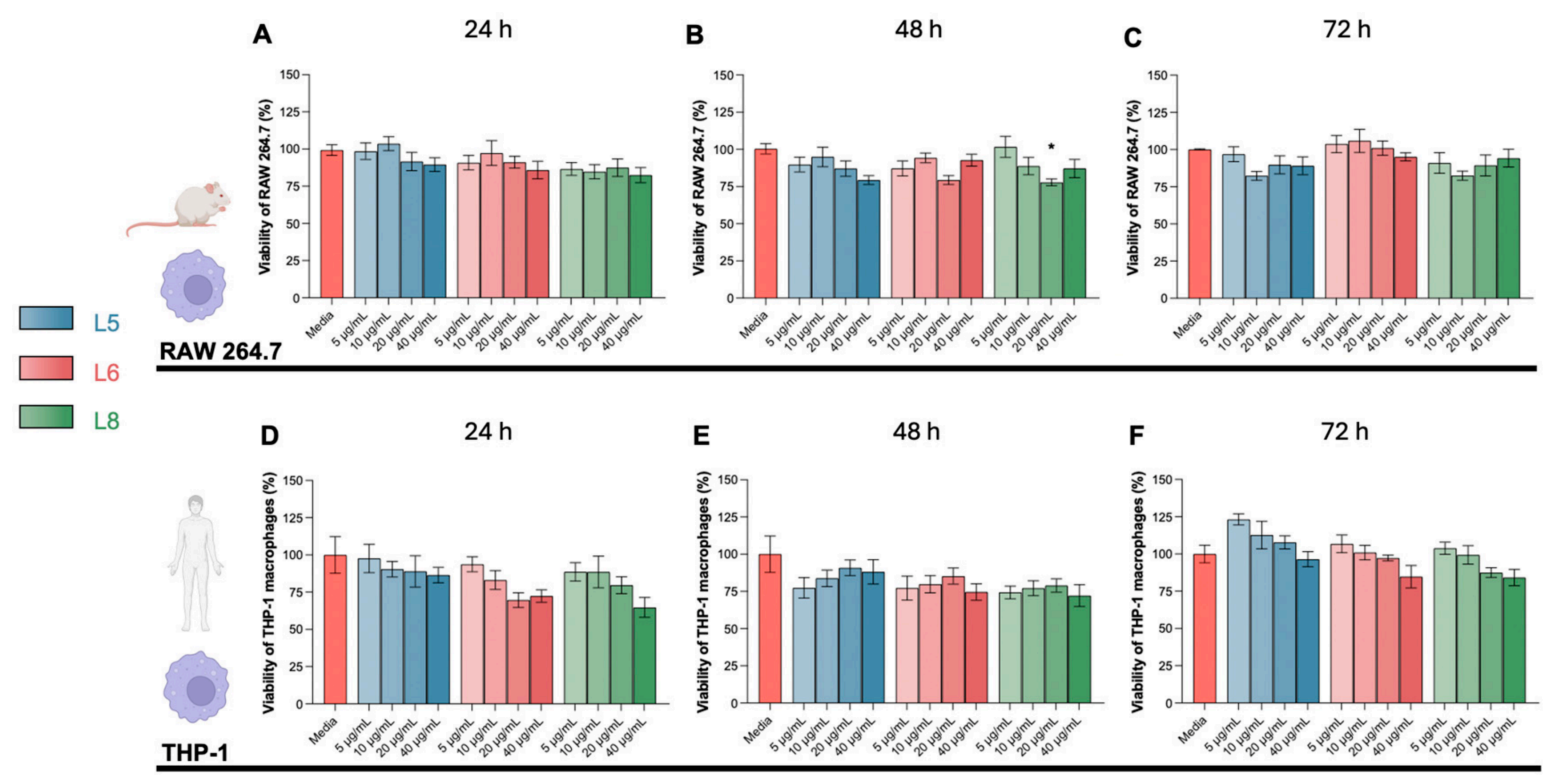
Viability of RAW 264.7 and THP-1 macrophages exposed to *P. lutzii* peptides. Murine RAW 264.7 (**A**–**C**) and THP-1 (**D**–**F**) macrophages were seeded in 96-well plates, and different concentrations of *P. lutzii* peptides were tested. Cells and peptides were incubated for 24 h, 48 h, and 72 h, respectively as shown in the figure. Controls of cells with only media without peptides were used. Graphic bars show the values of mean and error bars indicate standard error of the mean. Statistical analyses were performed using Kruskal–Wallis one-way ANOVA test compared with control group without peptide, where * *p* < 0.05. Data represent three independent experiments (*n* = 9). Representative elements created in BioRender (https://BioRender.com/f1m6lhp, accessed on 20 September 2026).

**Figure 3:**
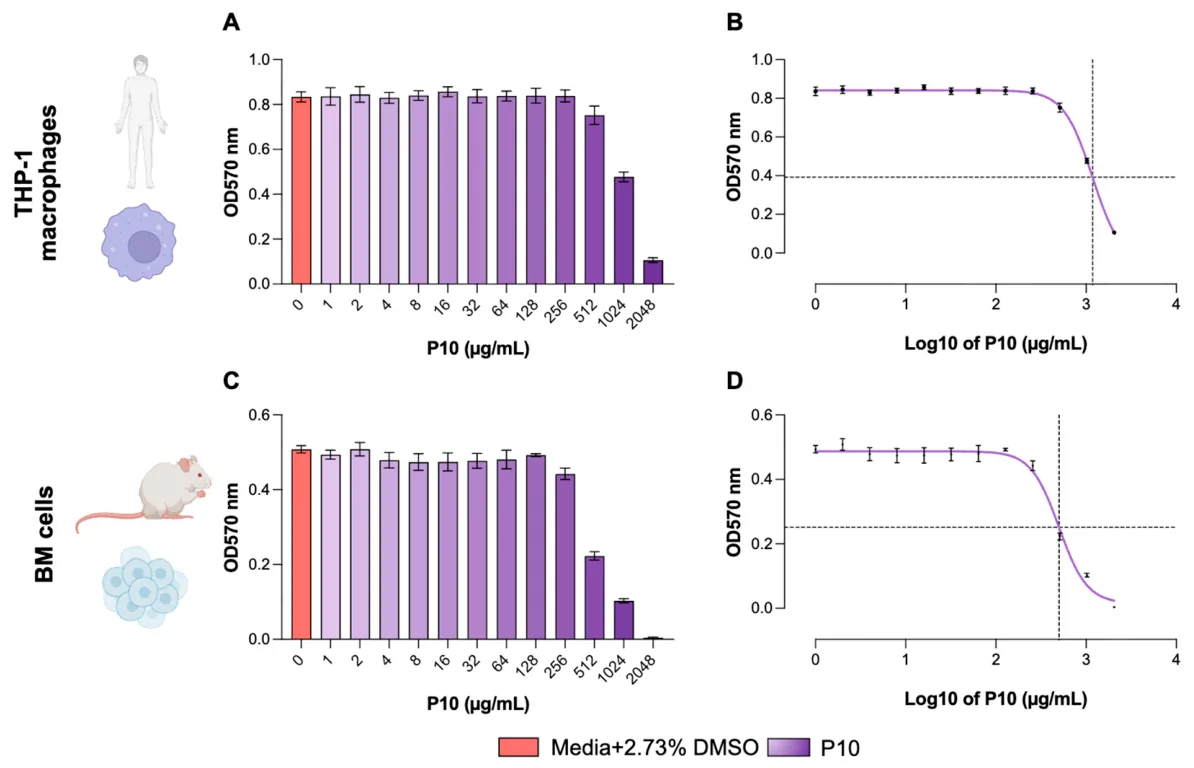
Determination of the IC50 of peptide P10 in THP-1 macrophages and murine bone marrow (BM) cells; (**A**,**B**) THP-1 macrophages and (**C**,**D**) murine BM cells were exposed to different concentrations of peptide P10. Cell viability was assessed, absorbance values were processed and plotted. Bar graphs show absorbance values, which directly reflect cell viability, with error bars representing the standard error of the mean. Nonlinear regression curves generated from the same dataset were used to calculate IC50 values, based on the log10 of P10 concentrations (*x*-axis) and absorbance values (*y*-axis). The point of intersection indicates the IC50. Data represents three independent experiments (*n* = 9). Representative elements created in BioRender (https://www.biorender.com/).

**Figure 4:**
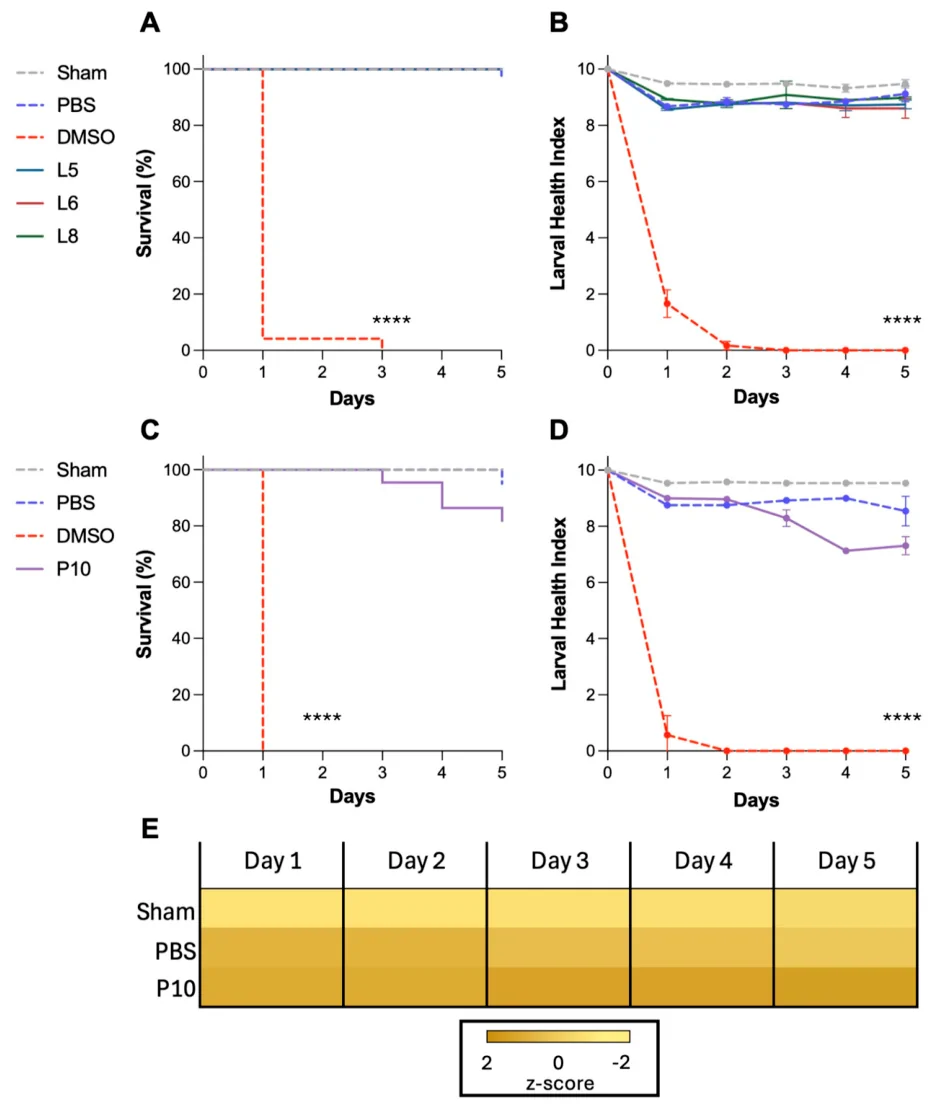
Larval survival and health index after injection with peptides. *G. mellonella* larvae were injected with *P. lutzii* peptides (2 mg/mL each) or P10 (10 mg/mL). Sham larvae (no injection) were used as untouched controls, PBS as injection control, and DMSO as a known toxic control. Larvae were monitored for 5 days. (**A**) Survival of larvae injected with *P. lutzii* peptides; (**B**) Larval Health Index (LHI) scores for *P. lutzii* peptides; (**C**) survival of larvae injected with P10; (**D**) LHI scores for P10; (**E**) LHI data were converted into a melanization index and normalized using z-score, presented as a heatmap for each condition across time. In Kaplan–Meier curves, overlapping lines at 100% may not be visible; XY plots show mean scores with error bars indicating standard deviation. Survival curves were analyzed using the Log-rank (Mantel–Cox) test, and LHI data by one-way ANOVA (**** *p* < 0.0001). Data represent three independent experiments for *P. lutzii* peptides (*n* = 40) and two for P10 (*n* = 20).

**Figure 5:**
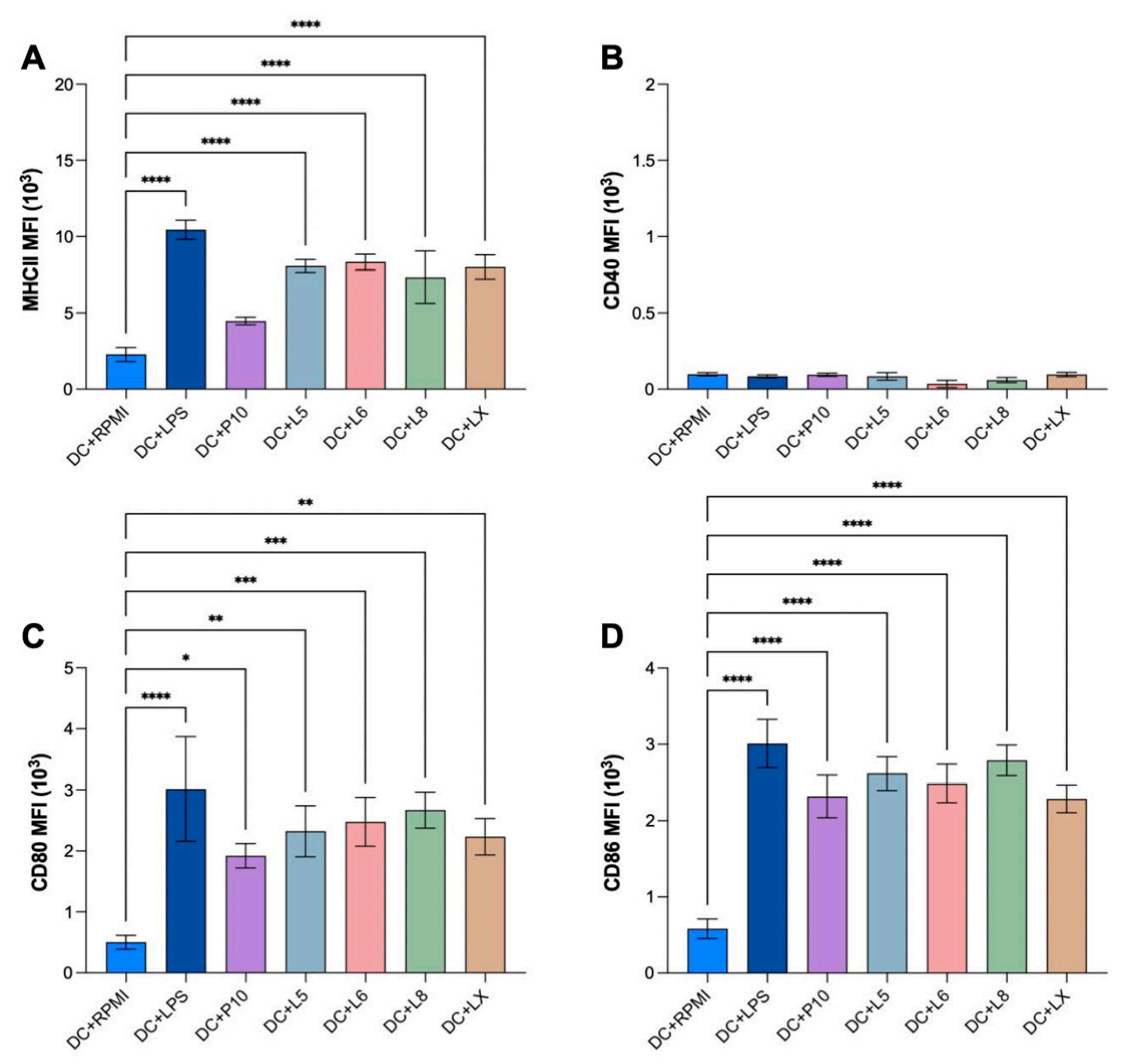
Expression of markers of murine bmDCs exposed to the peptides with 6 h incubation. The activation profile of bone marrow-derived dendritic cells (bmDCs) following peptide pulsing with 6 h of incubation, assessed by measuring the (**A**) MHC-II, (**B**) CD40, (**C**) CD80, and (**D**) CD86 molecules expressed on the cell surface. Graphs show the median fluorescence intensity (MFI), with error bars representing the standard deviation, where * *p* < 0.05, ** *p* < 0.01, *** *p* < 0.001, and **** *p* < 0.0001 compared with other groups. Statistical analyses were performed using Tukey’s multiple comparisons one-way ANOVA test. Data are representative of three independent experiments (*n* = 3).

**Figure 6:**
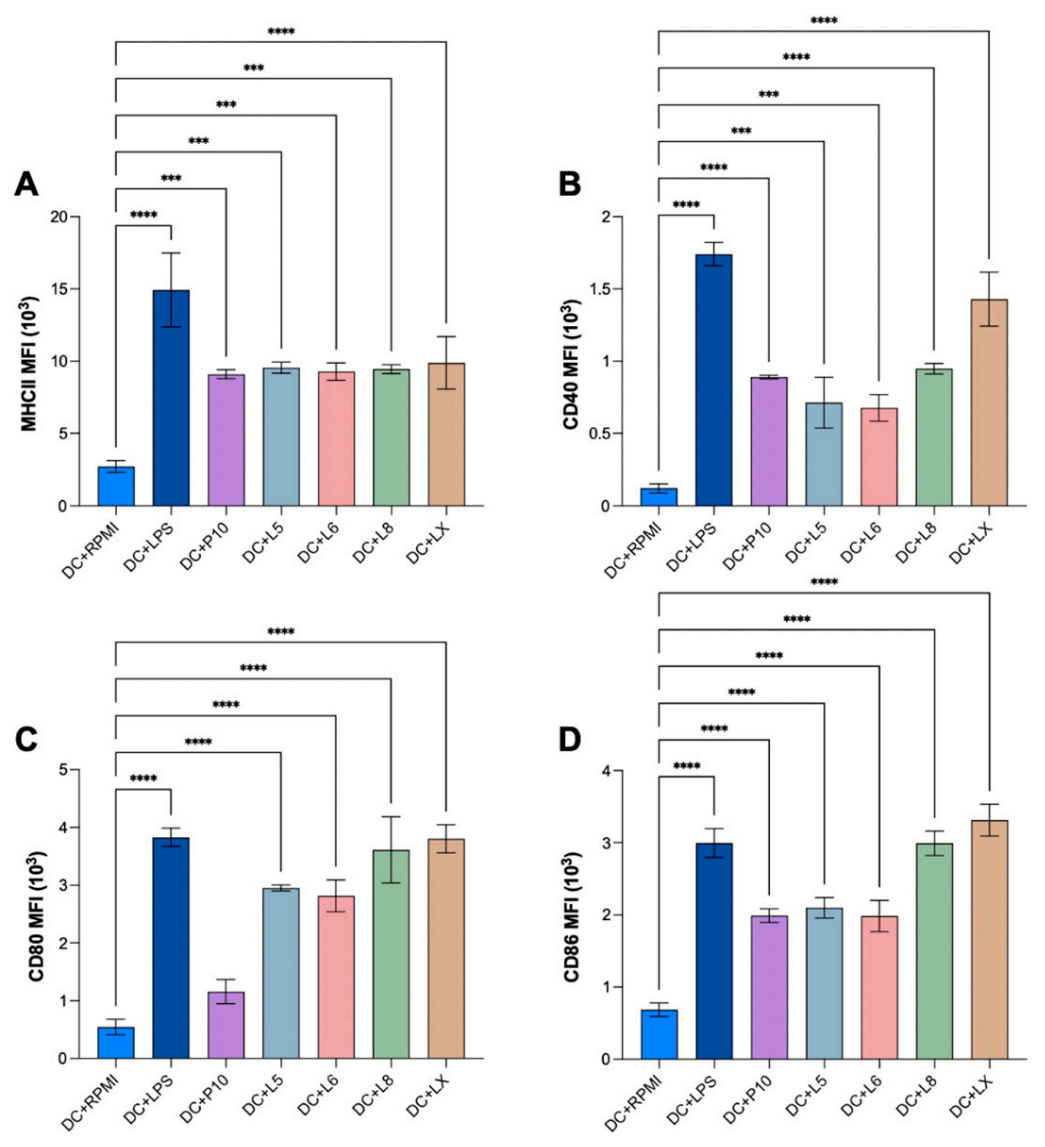
Expression of markers of murine bmDCs exposed to the peptides with 22 h incubation. The activation profile of bone marrow-derived dendritic cells (bmDCs) following peptide pulsing with 22 h of incubation, assessed by measuring the (**A**) MHC-II, (**B**) CD40, (**C**) CD80, and (**D**) CD86 molecules expressed on the cell surface. Graphs show the median fluorescence intensity (MFI), with error bars representing the standard deviation, where *** *p* < 0.001, and **** *p* < 0.0001 compared with other groups. Statistical analyses were performed using Tukey’s multiple comparisons one-way ANOVA test. Data are representative of six independent experiments (*n* = 6).

**Figure 7:**
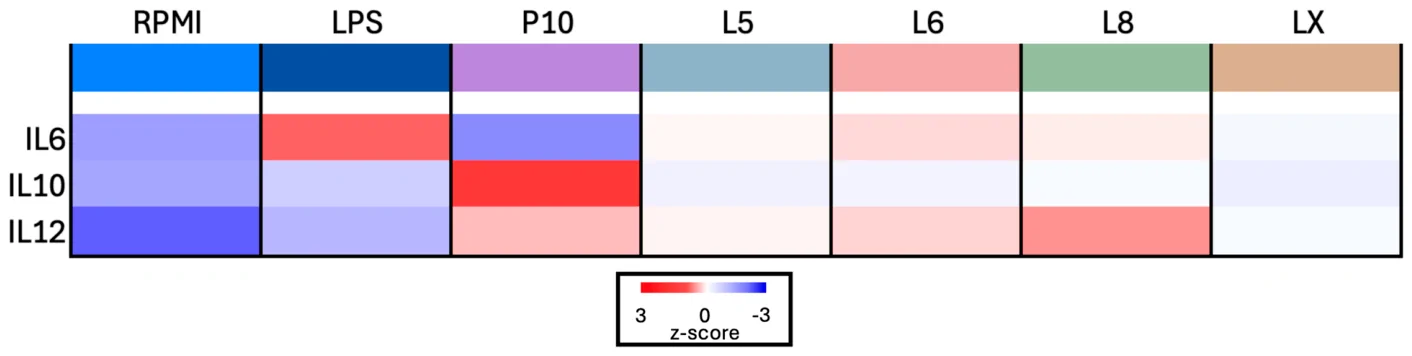
Heatmap of cytokines quantified from bmDCs supernatant with 22 h of pulsing with peptides. Supernatants from bmDC activation assays were collected and analyzed for cytokine production using ELISA kits. The heatmap represents cytokine levels detected in the supernatants of cells incubated with peptides. Data were normalized using z-scores, and the color gradient was generated with a Python script. The upper row indicates the color assigned to each peptide for colorimetric representation only. Results represent three independent experiments (*n* = 3).

**Figure 8:**
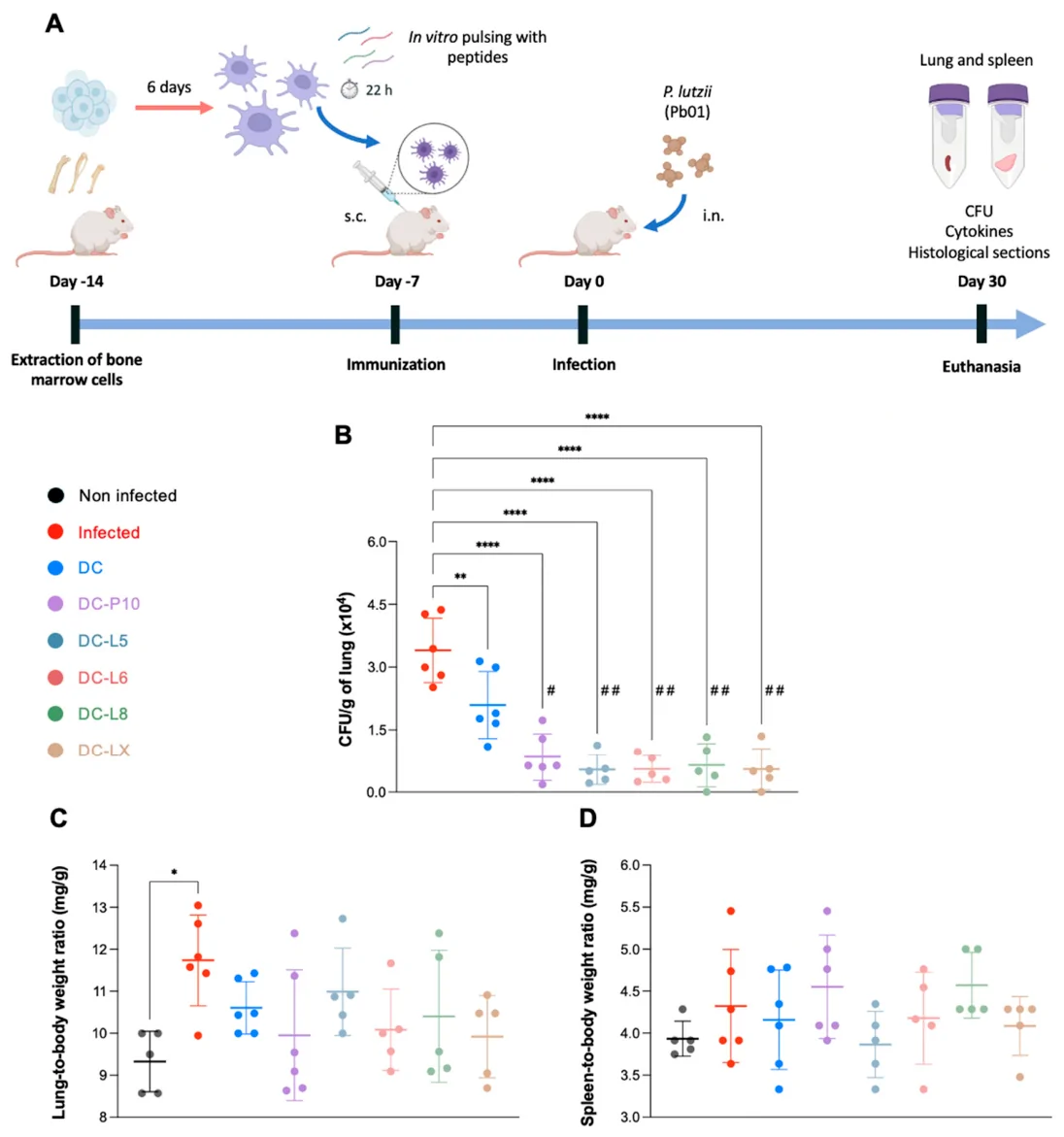
In vivo approach and fungal burden after peptide-pulsed bmDCs prophylactic immunization. (**A**) Schematic representation of the prophylactic bmDC immunization and infection timeline. Representative elements were created with BioRender (https://www.biorender.com/); (**B**) pulmonary fungal burden determined by colony-forming units (CFUs). Lung homogenates were plated and incubated at 35 °C; (**C**) lung-to-body weight ratio; (**D**) spleen-to-body weight ratio; dots represent individual animals, and bars indicate mean with standard deviation. Statistical significance was determined using one-way ANOVA followed by Tukey’s multiple comparisons test. Statistical comparisons are indicated as * *p* < 0.05, ** *p* < 0.01 and **** *p* < 0.0001 compared with the infected group, and # *p* < 0.05 and ## *p* < 0.01 compared with the DC group. Sample sizes vary between groups and are indicated in the figure (according to the number of dots in each group).

**Figure 9:**
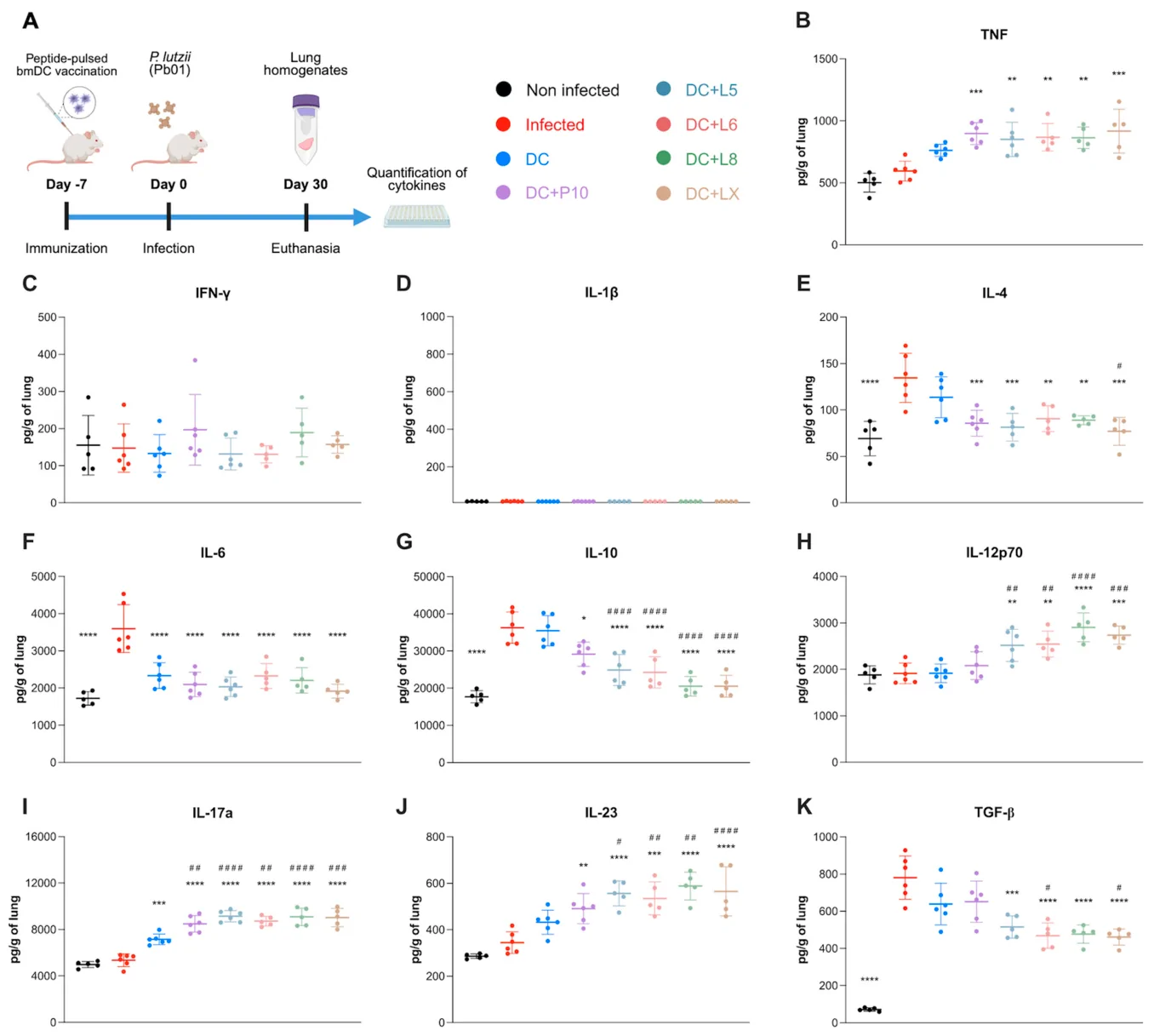
Cytokines levels in the lungs of mice. (**A**) Brief schematic illustration of the prophylactic bmDC immunization and infection timeline. After immunization and infection, mice were euthanized, and organs were collected and processed; (**B**–**K**) cytokines from lung homogenates were quantified by ELISA kits; graph bars show the mean with standard deviation, and dots represent individual animals. Statistical significance is indicated as * *p* < 0.05, ** *p* < 0.01, *** *p* < 0.001, and **** *p* < 0.0001 when compared to the infected group, and # *p* < 0.05, ## *p* < 0.01, ### *p* < 0.001, and #### *p* < 0.0001 when compared to the DC group. Analyses were performed using one-way ANOVA with Tukey’s multiple comparisons test. Sample sizes vary between groups and are indicated in the figure (according to the number of dots in each group).

**Figure 10:**
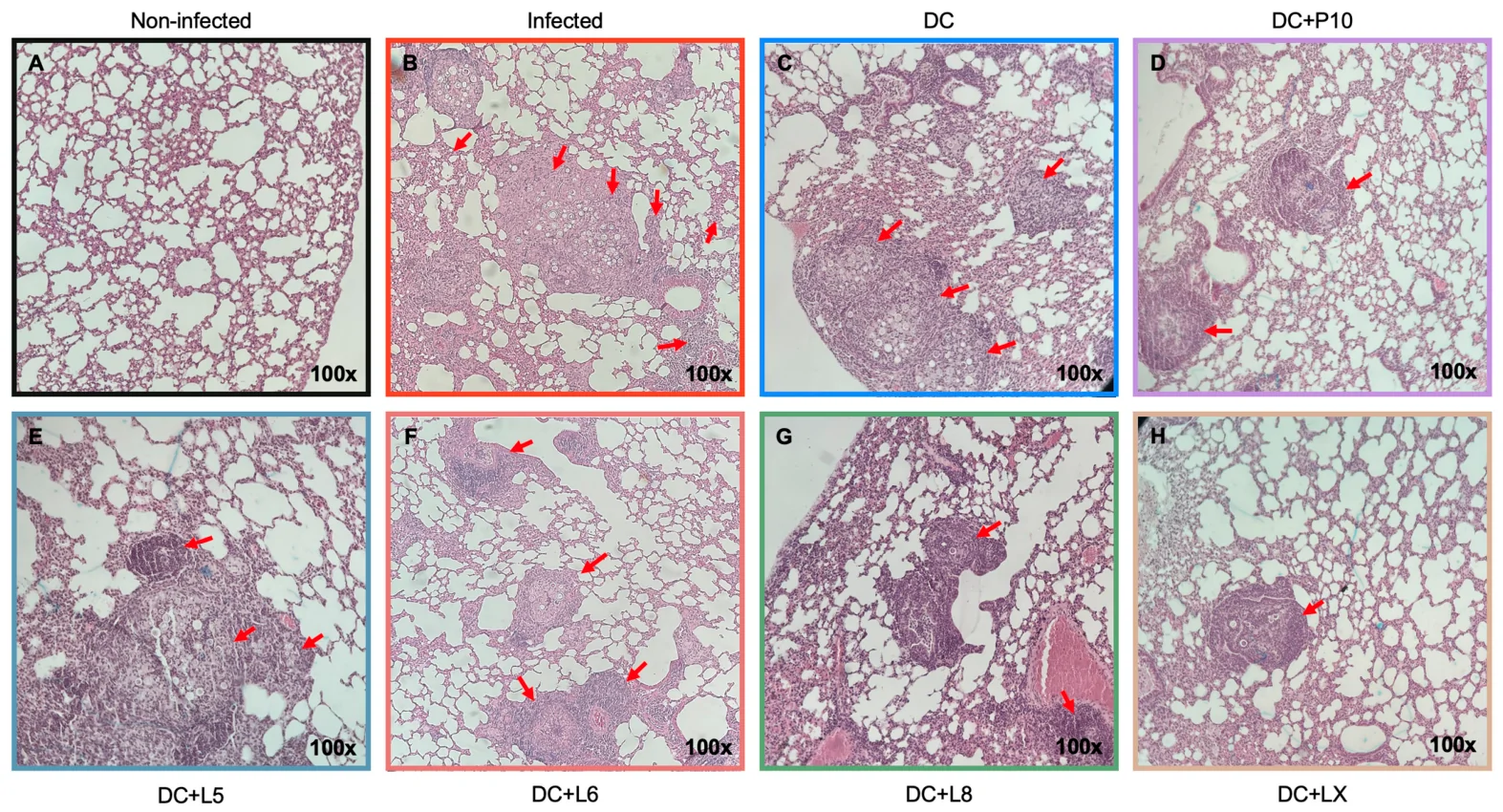
Lung tissues were collected, sectioned, stained with hematoxylin and eosin (HE), and analyzed by conventional light microscopy. Red arrows indicate granulomas. (**A**) Non-infected control animals showing preserved pulmonary architecture with normal alveolar spaces; (**B**) infected animals presenting inflammatory infiltrates and granulomatous lesions with poorly organized structure (red arrows); (**C**) animals immunized with dendritic cells (DCs) displaying inflammatory infiltrates and granuloma formation; (**D**) DC + P10 group showing organized granulomatous lesions; (**E**) DC + L5 group presenting compact inflammatory infiltrates with granuloma formation; (**F**) DC + L6 group showing granulomatous lesions distributed within the pulmonary parenchyma; (**G**) DC + L8 group displaying inflammatory infiltrates with compact granuloma formation; (**H**) DC + LX group presenting organized granulomatous structures; representative fields were acquired at 100× magnification.

**Table 1:** Amino acids sequence and relevant information of each peptide.

Name	Amino Acid Sequence	Protein Origin	Species of Origin	Reference
L5	DAGTIAGLNVLR	Hsp70	*P. lutzii*	[42]
L6	QHPSELETAIAGALSDLEANTPDLK	40S ribosomal protein S7	*P. lutzii*	[42]
L8	ITVTSQSAFDAQFNR	H15 domain-containing protein	*P. lutzii*	[42]
P10	QTLIAIHTLAIRYAN	gp43	*P. brasiliensis*	[33,34,43]

Note: gp43, glycoprotein 43 kDa; H15, histone 15; Hsp, heat-shock protein.

**Table 2:** In silico physicochemical properties of the peptides.

Name	Molecular Weight (g/mol)	pI (pH)	Net Charge at pH 7	Extinction Coefficient (M^−1^ cm^−1^)	Instability Index	Estimated Half-Time
L5	1199.37	6.68	0	0	18.14 (stable)	• 1.1 h (mammalian reticulocytes, in vitro); • 3 min (yeast, in vivo); • >10 h (*E. coli*, in vivo).
L6	2620.85	3.79	−3.9	0	10.65 (stable)	• 0.8 h (mammalian reticulocytes, in vitro); • 10 min (yeast, in vivo); • 10 h (*E. coli*, in vivo).
L8	1684.83	6.65	0	0	49.01 (unstable)	• 20 h (mammalian reticulocytes, in vitro); • 30 min (yeast, in vivo); • >10 h (*E. coli*, in vivo).
P10	1698.00	9.55	1.09	1280	23.29 (stable)	• 0.8 h (mammalian reticulocytes, in vitro); • 10 min (yeast, in vivo); • 10 h (*E. coli*, in vivo).
Database	PepCalc	PepCalc	Bachem	PepCalc	Expasy ProtParam	Expasy ProtParam

Notes: pI, Isoeletric point; M^−1^·cm^−1^, molar extinction coefficient units; h, hours; min, minutes. Instability index values > 40 indicate instability.

**Table 3:** Solubility of peptides with in silico predictions and experimental verification.

Name	Average Hydrophobicity	Estimated Solubility	Practical Solubility
L5	−0.17	Poor water solubility.	Successfully solubilized; no precipitates observed.
L6	0.27	Good water solubility.	Successfully solubilized; no precipitates observed.
L8	−0.19	Poor water solubility.	Successfully solubilized; no precipitates observed.
P10	−0.71	Poor water solubility.	Successfully solubilized (adding DMSO); no precipitates observed.
Database	Bachem	PepCalc	Practical experiment

Note: DMSO, dimethyl sulfoxide.

**Table 4:** Statistical results of the IC50 of peptide P10 in THP-1 human and BM murine cells.

Peptide	Cell Type	IC50 (Best-Fit Data)		95% CI	
		µg/mL	µmol/L	µg/mL	µmol/L
P10	THP-1 macrophages	1178.0	693.8	1046.0–1497.0	616.0–881.6
	mice BM cells	501.0	295.0	436.8–604.3	257.2–355.9

Note: 95% CI, 95% confidence interval; BM, bone marrow; IC50, half-maximal inhibitory concentration; THP-1, human monocytic leukemia cell line.

## Data Availability

The original contributions presented in this study are included in the article/Supplementary Material. Further inquiries can be directed to the corresponding authors.

## Supplementary Materials

The following supporting information can be downloaded at https://www.mdpi.com/article/10.3390/jof12100725/s1. Table S1: Detailed Statistical Analysis of Lung Fungal Burden.

## Abbreviations

The following abbreviations are used in this manuscript:

APC	Antigen-presenting cell
BM	Bone marrow
bmDC	Bone marrow-derived dendritic cell
CEUA	Ethics Committee on the Use of Animals
CFU	Colony-forming unit
CONCEA	National Council for the Control of Animal Experimentation
DC	Dendritic cell
DMSO	Dimethyl sulfoxide
FBS	Fetal bovine serum
gp43	43 kDa glycoprotein
H15	Histone H15
H&E	Hematoxylin and eosin
HPLC	High-performance liquid chromatography
Hsp70	Heat-shock protein 70
IC50	Half-maximal inhibitory concentration
LHI	Larval Health Index
LPS	Lipopolysaccharide
MS	Mass spectrometry
MTT	3-(4,5-Dimethylthiazol-2-yl)-2,5-diphenyltetrazolium bromide
PCM	Paracoccidioidomycosis
PMA	Phorbol 12-myristate 13-acetate
WHO	World Health Organization
